# The British Journals, &c.: Surgery

**Published:** 1842-07

**Authors:** 


### SURGERY.

CASE OF ENLARGEMENT OF THE NOSE TREATED SUCCESSFULLY. The patient was a young lady, who had a peculiar enlargement of the nose, unaccompanied by pain or inconvenience, except that arising from the size. Dr. C. suspected it to be owing to deficient menstruation, for which he instituted constitutional treatment. His local application was as follows: "Taking a quantity of plaster of Paris, I made a mould of the nose, and whilst wet, I placed tapes in the plaster to secure it afterwards; the middle of one tape fastened to the mould was intended for securing it laterally by each end crossing the cheek on the same side, and tying together behind the neck; a second tape directed its course between the eyes over the centre of the os frontis, over the head, and secured to the first tape behind the neck; when sufficiently hard, the mould was removed, baked, and well seasoned with oil; when thus prepared it was replaced on the nose, and secured by the tapes so as to effect a gentle and equal pressure on the organ, the weight of the mould assisting, as it was made purposely rather thick, the lower part being left open to facilitate breathing. After wearing it in this manner a week, I found the mould much too large for the nose, and sat very loosely upon it. I was therefore certain the pressure had effected a considerable reduction in the size of the part affected: encouraged by this, a second mould was made on the reduced organ, which was accompanied with the same satisfactory results; a third, fourth, and fifth mould followed, when the nose had assumed its natural size and appearance. On comparing the last with the first mould, the contrast was very striking, and would scarcely have been believed by any person who had not witnessed the process: each mould was worn about a fortnight, with the exception of the first and last; the former about a week; the latter was advised to be worn longer, and relinquished by degrees; the constitutional treatment succeeded in effecting menstruation regularly, and in a sufficient quantity. The nose still remains its natural size. I think this plan might be applied with advantage in many cases; the effect of pressure in chronic enlargements is well known; it is only the novel way of employing it that deserves attention in this case."—Dr. CHARLES CLAY, *Lancet*, No. iii., *April* 16, 1842.

---

FIVE CASES OF STONE TREATED BY LITHOTRITY. The first patient, fifty-six years of age, required five sittings, seemed to have suffered very little from the operation, and was cured. The passage of the fragments of stone subsequent to the operations was not followed by any irritation. The second patient, aged thirty, required three sittings. In this case the pain attending the operation was trifling; but the first manipulation was followed by feverishness and inflammation of one testicle, which rendered a postponement of further proceedings necessary, from the 1st to the 18th of June; cure. In the third case, which was that of a female whose age is not stated, three sittings were required; cure. In the fourth case, that of a man sixty-three years of age, who had a stone measuring seven lines, the operation, performed on the 12th of October, was accompanied with little pain; but on the 16th the urine deposited a considerable quantity of viscid, transparent mucus, which gradually disappeared under the use of the oil of cubebs. The operation was repeated on the 7th and 25th of November, and the 5th, 19th, and 26th of December, without being again followed by catarrh of the bladder, or any other inconvenience; cure. But a year after, symptoms of stone reappeared, which were again removed after two lithotritical sittings; and the man being examined by three hospital surgeons, was found free from stone. The fifth was the only unsuccessful case. After lithotrity had been performed and fragments of stone removed, the bladder became so irritable as to make lithotomy necessary. This was performed, and three calculi as large as hazel-nuts, and numerous small, soft concretions, were removed. But fresh symptoms of stone have since manifested themselves. This patient was a man of seventy-two, but healthy.—Mr. TEALE, *Prov. Med. and Surg. Journal*, No. xxiv., *March* 12, 1842.

---

OPERATION FOR HARE-LIP PERFORMED ON A CHILD FOUR DAYS OLD. There was nothing remarkable connected with the operation, except the early age at which it was performed. The parts seem, moreover, to have united with unusual rapidity and completeness The author's reason for operating so soon was, that the mother was disagreeably affected by the appearance of the hare-lip in her child, and wished it removed as speedily as possible; but it is open for consideration whether, in every case, it may not be better to operate at a much earlier period than is usually done in such cases.—Dr. DAWSON, *Dublin Med. Press*, No. clxviii., *March* 23, 1842.

---

DRY GANGRENE OF THE ARM. John Silver, aged fifty-one, a stout, muscular man, whose constitution had become much impaired by free living, and constantly driving a night-coach to and from Exeter to this town, felt considerable pain in his left arm whilst performing the journey on a cold, rough night in the month of February, 1813. On the following morning the hand was found to be dark-coloured, cold, and shrivelled, which appearance, on further examination, extended to the elbow. He consulted an old practitioner, who recommended fomentations with mustard and horse radish, bark, wine, and brandy. These remedies produced no good effect, and the disease proceeded until it had nearly reached the shoulder-joint. At this time I saw him, and proposed amputation as the only thing to be done. It was refused, but ultimately the limb was removed immediately below the joint. The pulsation of the brachial artery was so feeble that it could scarcely be felt before the operation; and when the vessel was divided it bled very languidly, the discharge being so trifling that it seemed scarcely necessary to apply a ligature. The wound slowly, but never completely, healed, and he recovered sufficiently to enable him to go about for some months, when he died.

The progress of this disease resembled gangrena senilis, many examples of which I have seen affecting the lower extremity in old people; but this is the only case in which I have observed the upper extremity affected. As no post-mortem examination could be obtained, it was impossible to ascertain the condition of the blood-vessels, which would have been very desirable."—JONATHAN TOOGOOD, Esq., *Prov. Med. and Surg. Journal*, No. xxiv., *March* 12, 1842.

---

AIR-DOUCHE OF THE EUSTACHIAN TUBE. This paper is violently dissuasive of air-injections of the Eustachian tube. The author seems to think that the injection is not actually effected in a great many cases in which it is pretended by the operator and imagined by the patient to have been done. Among the disastrous effects which have resulted from attempts to perform the operation, or from its actual performance, he enumerates inflammation of the throat and tube itself, as well as of the tympanum, so severe as sometimes to terminate in suppuration; emphysema caused by laceration of the mucous membrane of the Eustachian tube or posterior nares; rupture of the membranum tympani; deliquium so protracted as to threaten life; death. These last two formidable consequences the author considers are to be referred to the *direct pressure of air upon the brain*, in consequence of the enormous force sometimes exerted in injecting with air a narrowed Eustachian tube.

The author has found remarkable good effects from the use of iodine in conjunction with sarsaparilla, in the swelled tonsils succeeding the inflammatory sore throat of scarlatina, by which deafness is so often induced.—ANONYMOUS, *Medical Gazette*, No. xxxiii., *May* 6, 1842.

---

OPERATION FOR ARTIFICIAL ANUS. The object of this paper is to show the superiority of Amussat's method of operation over that of Callisen, since by the latter the peritoneum is twice opened; by the former, that membrane is left untouched. The author details a very interesting case of Amussat's, at which he himself was present. It occurred on the 20th of January last. A child, within a few hours of its birth, was taken to M. Larrey on account of some impediment to the exit of the fæces. A cul de sac, about an inch and a half from the anus, was detected. M Larrey having tried without success the introduction of the catheter, plunged a trocar into the cul de sac (as was supposed), but no meconium followed the withdrawal of the instrument. The child was brought to Amussat; it was now forty-eight hours old. The abdomen was hard and distended, and the child's face dusky. It had vomited frequently. Dr. Amussat, on examination, was led to believe that about two inches from the anus there existed an interruption of the rectum, the caliber of the gut being at this point totally obliterated; and he was of opinion that it was totally impracticable to form an artificial anus either in the anal or coccygeal regions, but that an incision into the colon, in the left lumbar region, afforded the only chance of life to the child. Amussat's mode of operation has already been described in this Journal.

The case, up till the time of report (four weeks from the date of the operation), has done well. Tepid injections are administered every twenty-four hours, and the fæces escape readily through the artificial anus. A small tent is kept constantly in the aperture, which prevents the closure of it.—Mr. Parrott, *Medical Gazette*, No. xxvii., *March* 25, 1842.

---

Operation for Artificial Anus. "Amussat," remarks Mr. Teale, "has shown that the failure of the operation on the dead subject was owing to the intestine being empty, and that in such cases as require the formation of an artificial anus, the colon is greatly distended, in which condition the layers of the peritoneum, forming its imperfect mesentery, are so far separated as to admit of the intestine being reached without opening the peritoneum. He has farther introduced an important modification of the operation of Callisen, by adopting the transverse instead of the vertical incision of the muscles." The operation, as performed by Mr. Teale, was as follows. The child seems to have died the sixth day after the operation: "The patient being placed upon a table, on the right side, with the face and abdomen inclining downwards, Mr. Teale made an incision through the integuments four inches and a half in length, extending forwards from the outer edge of the sacro-lumbalis and longissimus dorsi muscles, midway between the lower ribs and the crest of the ilium, nearly parallel to the latter. The different layers of aponeurosis and muscle having been divided in succession upon a director, a considerable mass of fat was forcibly protruded at the wound. By the finger the packets of fat were detached, and the posterior surface of the colon was very readily felt, extremely tense and elastic, and was soon exposed to view, its pale blue tint and translucent aspect contrasting strongly with the opaque white fat in the neighbourhood. Two temporary ligatures were passed through the muscular and mucous coats of the colon, and a considerable quantity of air escaped through the punctures, which so far diminished the tension of the intestine, that Mr. Teale was enabled to pinch up a fold of it, and to open it with the scalpel, after which there was a profuse discharge of air and liquid fæces. The opening in the intestine was further dilated in a vertical direction by a probe-pointed bistoury to such an extent as to allow of the introduction of three fingers. The edges of the intestinal aperture were then fixed to the external wound by four points of suture, and the wound of the integuments was united by two twisted sutures. The operation being completed, Mr. Teale and other surgeons present introduced the fore-finger into the artificial anus, and ascertained that the colon immediately below the aperture formed a thickened corrugated pouch, from which no opening into the lower part of the intestine could be detected without instituting a more tedious search than was considered justifiable. The intestinal tunics forming this pouch, although much thickened and firmer than natural, did not communicate to the touch the indurated feeling of scirrhus. On passing the finger upwards into the descending colon, it was found to be capacious, its coats thin and elastic, possessing a perfectly healthy structure."—Mr. Teale, *Prov. Med. and Surg. Journal*, No. xxv., *March* 19, 1842.

Reduction of a Dislocation of the Lower Jaw, 98 Days after the Occurrence of the Accident. There is nothing peculiar in this case, except the length of time which had elapsed between the occurrence of the dislocation and its reduction: and the author's principal object in publishing it, is to encourage other surgeons to undertake the operation, even under the most discouraging circumstances.—Dr. Donovan, *Dublin Med. Press*, No. clxxvii. *May* 25, 1842.

---

The Seton in Chronic Disease of the Brain. A farmer, sixty-four years of age, was seized with fever and acute inflammation of the brain, in consequence of sleeping on the damp grass. These seemed to have been treated in an active and judicious manner, yet an obtuse pain in the head remained, with at first slight mental obscuration; but which deepened, at length, into perfect childishness and idiocy. His bodily strength was also greatly impaired.

Dr. B. established a seton in the nape of the neck. In ten days there were faint traces of returning intelligence, and in five weeks the patient became again a useful member of society, and enjoys at present complete bodily and mental sanity.—Dr. Barton, *Lancet*, No. ix. *May* 28, 1842.

---

Subconjunctival Dislocation of the Lens. William Weavers, aged sixty-four, received a violent blow on the left eye, from a man's fist, more than five weeks since, which caused at the time severe pain and swelling, and loss of vision.

March 8th, 1841.—The upper part of the cornea had now become opaque, sloughy, and ulcerated; but the centre remained sufficiently transparent to allow of the examination of the pupil, which was contracted, fixed, and nearly filled with pus. At the upper part of the globe, a short distance behind the corneal and sclerotic junction, there was a circumscribed semi-transparent tumour of the conjunctiva, which both Mr. Barton and myself considered to be caused by the dislocated lens. I therefore divided the conjunctiva with a cornea knife, and extracted the lens inclosed in the unruptured capsule, both structures being perfectly transparent. I also punctured the lower part of the cornea, to relieve the eyeball from the distension occasioned by internal suppuration. He suffered much less after the removal of the lens, all the inflammatory symptoms subsided, and the eye gradually sank into a state of atrophy.—Mr. Hunt, *Med. Gazette*, No. xxxv. *May* 20, 1842.

---

Rare Fracture of the Neck of the Thigh-bone. The following fracture of the femur has not often been noticed by surgical writers. The following was the state of the patient when seen by Mr. B. He was, and had been, from the time of the receipt of the accident, unable to bear upon the affected limb, the attempt to do so causing intolerable pain. He could, however, with the assistance of his hands in lifting it, flex it to a considerable extent upon the trunk, while lying on his back in bed, without experiencing any particular uneasiness in the part. The affected limb had become shortened to the extent of about an inch, and was everted, but it was easily rolled inwards into its natural position. It was, however, impossible, or at least difficult, without great violence, to extend the limb so as to make it appear of the same length as the other sound one. There was an extensive ecchymosis of the integument round the joint, involving the skin of the nates and scrotum, of an extremely dark purple colour, like what is usually observed to follow sprains in very old persons. The limb could be rotated to some extent, both inwards and outwards, without much pain; this latter movement producing a grating or rubbing sensation, but very indistinct, and not at all resembling the feeling of crepitus commonly discernible in fractures of the neck of the bone. On pressing rather strongly with the fingers upon the posterior part of the trochanter major, a movement, as if this posterior portion of the trochanter had been detached from the shaft of the femur, was perceptible when the limb was in a quiescent state, but when it was flexed, or rotated, this feeling was not communicated, but only the dull rubbing or grinding sensation which I have described.

On account of the existing ecchymosis, and some little pain which the examination had produced, the limb was at first laid upon pillows in a flexed position, and allowed to remain so for two or three days; I then (the pain having left him) thought that I might use with advantage an apparatus constructed on the same principles as Boyer's, with an endless screw, having a foot-piece or shoe working upon it for the purpose of keeping up permanent extension; but as I found it was utterly impossible, by any mechanical means, to draw out the shortened limb to its natural length, without injurious compression of the perineum by the padded girth, and as he felt otherwise very uneasy from its application, it was relinquished, and Amesbury's double-inclined plane was substituted in its stead; he, however, soon became weary even of this, and he was ultimately placed, without any restriction of the limb (with the exception of firmly binding it to the other to prevent eversion,) upon one of the double-inclined plane beds of the hospital, with which he expressed himself as quite satisfied, and felt comfortable and easy.

The man sank from bronchial inflammation and diarrhœa, and the results of the post-mortem examination of the limb are thus recorded: The subcutaneous ecchymosis had disappeared before his death, but a considerable quantity of blood had remained diffused among the muscles surrounding the fractured portion of the bone. The capsular ligament was entire, but it was somewhat thicker than natural, owing probably to the effects of the rheumatic inflammation in the joint to which he had been subject. On cutting through the capsule, the neck of the bone, which was somewhat thicker and shorter than common, was found to be uninjured, the fracture not having in the slightest degree encroached upon this portion of the bone. The head and cervix of the bone had been obliquely separated from the shaft, just at the root of the trochanter minor, and had passed downwards into the cancellous structure of the bone, which they were enabled to do, owing to the posterior portion of the trochanter major having been broken off, opening as it were to receive them.

The fracture of the trochanter major commenced exactly in the centre of its upper part, and, passing downwards through the length of the trochanter, the fissure wound inwards, and terminated at a little distance below the trochanter minor, which was included in the fractured piece.

It was the movement of this portion of the bone, which I felt during the lifetime of the patient, when I pressed my fingers strongly on the posterior part of the trochanter, while the limb was still and straight, but which was not observed when it was flexed, owing, as I now found, to the fractured neck passing (when flexure was attempted after death) lower down into the cancellous structure, and wedging out, and making immovable this fractured portion, the movement of which was before so clearly distinguished.

On partially removing the fractured portion of the bone, the cancellous structure was found to have been hollowed out, as if by the attrition of the fractured neck within it, which had been caused by the patient injudiciously moving the limb, which had at the same time interrupted the reparative process, and a complete hollow had been formed at this part. The spongy texture itself was not more vascular than is commonly seen after a fracture has occurred a few days. The outer shell of the bone was, however, remarkably thin, and the medullary cavity, for some distance down the shaft, was somewhat larger than is usual in persons even of a very advanced age.

I could now discover what had prevented the extension of the limb; owing to this excavated condition of the cancellated interior of the bone, the upper and fore part of the trochanter major, which had not been broken, had been drawn inwards by the muscles inserted at its root, and had closed upon the end of the fractured neck after it had passed downwards, below it, and on endeavouring to extend the limb after death, the neck of the bone catching under this firm portion of the trochanter prevented the extension of the limb, and I found that I was unable, even after the muscles were removed, to cause the trochanter to pass over the fractured end of the cervix without applying more force than would have been justifiable in the lifetime of the patient. The thorax was not examined.—Mr. BULLEY, *Prov. Med. and Surg. Journal,* No. iv. *April* 30, 1842.

Paracentesis of the Abdomen with the common grooved Needle. As paracentesis of the thorax, (observes Dr. P.) by means of a trocar, is much more painful than the introduction of the needle, and is sometimes followed by troublesome results, I determined to try for the future, whether the same success would follow the use of the latter instrument. In many instances this was found to be the case, but occasionally the fluid was too thick to run off through so small a passage. This was obviated by getting a needle made with a somewhat larger groove. Such a needle has been used with perfect success in every case that has fallen under my observation during the last two years. The pain is so slight, that a patient who has once experienced it totally disregards it. The following remark of Dr. P. is worthy of serious attention: I am persuaded that the practice of deferring paracentesis in the former disease (ascites) till all the other means, many of which are very exhausting ones, have been long tried and have failed, is one principal cause of the frequent return of the effusion. The use of the needle affords so much greater facility that the operation will cease to be dreaded, and will be performed more easily, and if so, with greater success.—Dr. Prichard, of Bristol. *Medical Gazette*, No. xxvii. *March* 25, 1842.

---

Traumatic Tetanus: Cure. This was a decided case. The patient had wounded a finger with a straw-cutting instrument, on the 5th of February. On the night of the 21st, he slept in an exposed situation; and on the 25th, the author found him complaining of violent spasmodic pain at the epigastrium, with great difficulty of breathing; stiffness in the muscles of the neck, and inability to open the mouth; the pulse full; 120. He was bled to ℥xx, and on the succeeding day to ℥xxx, which relieved, but only temporarily. He had two grains of opium every three hours. On Sunday, he was in a state of complete opisthotonos, and intoxicated from the effect of the opium; but suffering little or no pain, when undisturbed; he could breathe, talk, and swallow easily. On Monday, his circumstances were exactly the same. Mr. H. found him in precisely the same position as he had left him in on the preceding day—that is, his body resting on his heels and head. He was again bled to ℥xvj. On the night of March 2, he had profuse perspiration, with great relief. The opisthotonos was so far gone as to allow the patient to turn on his side. At the date of report he was nearly well.—Mr. Higgins, *Lancet*, No. v. *April* 30, 1842.

[This case is extremely creditable to Mr. Higgins. Another case is reported in the Provincial Medical and Surgical Journal for April 30, 1842, No. iv., which was successfully treated by sesqui carbonate of iron. In this case there was complete emprosthotonos, with incomplete trismus.]

---

Spontaneous Obliteration of the Axillary Artery. A lady aborted on the 7th November, 1831. On the afternoon of the 8th, she felt a sensation at the extremities of the fingers, as if they were scorched. The integuments at the points felt hard, looked white, and were painful, so that she apprehended she was going to have whitloe in all her fingers. She awoke on the following morning from a disturbed sleep, with intense pain and numbness of the whole arm, and almost total blindness. The left arm was now cold and insensible. The wrist and the tops of the fingers were growing discoloured: the ring-finger especially was becoming black. And now no pulsation could be felt in the axillary artery, or any of its branches, below the superior margin of the pectoralis major. This was on the 9th. On the 13th, the sense of feeling and temperature began slowly to return, though the arm still continued very painful; and the discoloration declined: but the ring-finger became black and hard at its extremity, and around the dry crust at that point appeared a line of separation, consisting of a slightly elevated circle, containing a minute quantity of pus. The top of this finger exfoliated, like a dry, hard, black, horn button, nine months after the obliteration of the vessel. So did the integumental extremities of the thumb, first, second, and last fingers. The author thinks the obliteration of the artery is to be accounted for from sudden spontaneous rupture of the internal coat of the artery.—Dr. Oke, *Provincial Medical and Surgical Journal*, No. iii. *April* 23, 1842.

CASE OF LARGE CALCULUS IN A FEMALE CHILD THREE YEARS AND A HALF OLD. After various fruitless attempts at extraction, by dilatation of the urethra, and by lithotritizing, the lateral operation was performed, and a lithic-acid stone was extracted, measuring an inch and a quarter in length, seven-eighths of an inch in width, two inches and a half in circumference, and weighing two drachms, forty grains. The case did well.—Mr. GRANTHAM, *Med. Gaz.* No. xxiv. *March* 4, 1842.

---

PENETRATING WOUND OF BOTH LUNGS: RECOVERY. A policeman, aged twenty-two, was stabbed on the right side, about four or five inches below and in a direct line from the axilla; the wound was rather more than an inch in width, and was between the seventh and eighth ribs. On the left side there was a corresponding wound, also between the seventh and eighth ribs, but higher up and more posterior, situated almost in a direct line from the lower angle of the scapula. There was emphysema on both sides, and air was passing freely through the wound on the right side. The wounds were closed with plaster, and the patient, who was cold, had some tea. The man was wounded at four o'clock in the morning, and Mr. R. saw him between five and six. At nine, there were symptoms of reaction, for which venesection to ℥xx, and spermaceti and ipecacuan wine were ordered. At three o'clock of the same day he was bled to ℥xxxiv, and at nine o'clock of the same evening to ℥xxx. On the following day, the air did not pass so freely out and in of the wounded apertures, on the dressings being removed. He was bled to a small extent the same day. He continued to improve from this time, and eventually recovered. Adhesion of the pleura, to a considerable extent, had taken place on the right side; the state of parts, on the left, could be less easily ascertained. Mr. RUDDOCK, *Prov. Med. and Surg. Journal*, No. xxvii. *April* 2, 1842.

---

SUCCESSFUL CASE OF TRACHEOTOMY. The patient was a man of twenty-four years of age, and the symptoms were, of course, urgent. In performing the operation, Mr. A. attempted to use the tracheotomy trocar, having no other tube but the canula fitted to it, but he could not make the point of the stilette enter the trachea, as the cartilages bent under the pressure. He therefore opened the trachea with the knife, but, ere this could be done, the man was insensible. He, however, soon rallied, and a cure was effected. About eight months after the operation (the date of the last report) he was still obliged to employ the canula, as the want of it for more than a minute or two caused distressed breathing.—Mr. ALFORD, *Prov. Med. and Surg. Journal*, No. xxvi. *March* 26, 1842.

